# Amelioration of Lupus Nephritis by Serum Amyloid P Component Gene Therapy with Distinct Mechanisms Varied from Different Stage of the Disease

**DOI:** 10.1371/journal.pone.0022659

**Published:** 2011-07-25

**Authors:** Weijuan Zhang, Jin Wu, Bin Qiao, Wei Xu, Sidong Xiong

**Affiliations:** 1 Institute for Immunobiology and Department of Immunology, Shanghai Medical College, Fudan University, Shanghai, People's Republic of China; 2 Institutes of Biology and Medical Sciences, Soochow University, Suzhou, People's Republic of China; Institut Jacques Monod, France

## Abstract

**Background:**

Our previous study revealed that administration of syngeneic female BALB/c mice with excessive self activated lymphocyte-derived DNA (ALD-DNA) could induce systemic lupus erythematosus (SLE) disease, indicating that overload of self-DNA might exceed normal clearance ability and comprise the major source of autoantigens in lupus mice. Serum amyloid P component (SAP), an acute-phase serum protein with binding reactivity to DNA in mice, was proved to promote the clearance of free DNA and prevent mice against self-antigen induced autoimmune response. It is reasonable to hypothesize that SAP treatment might contribute to alleviation of SLE disease, whereas its role in ALD-DNA-induced lupus nephritis is not fully understood.

**Methodology/Principal Findings:**

The ratios of SAP to DNA significantly decreased and were negatively correlated with the titers of anti-dsDNA antibodies in ALD-DNA-induced lupus mice, indicating SAP was relatively insufficient in lupus mice. Herein a pcDNA3-SAP plasmid (pSAP) was genetically constructed and intramuscularly injected into BALB/c mice. It was found that SAP protein purified from the serum of pSAP-treated mice bound efficiently to ALD-DNA and inhibited ALD-DNA-mediated innate immune response *in vitro*. Treatment of ALD-DNA-induced lupus mice with pSAP in the early stage of SLE disease with the onset of proteinuria reversed lupus nephritis via decreasing anti-dsDNA autoantibody production and immune complex (IC) deposition. Further administration of pSAP in the late stage of SLE disease that had established lupus nephritis alleviated proteinuria and ameliorated lupus nephritis. This therapeutic effect of SAP was not only attributable to the decreased levels of anti-dsDNA autoantibodies, but also associated with the decreased infiltration of lymphocytes and the reduced production of inflammatory markers.

**Conclusion/Significance:**

These results suggest that SAP administration could effectively alleviated lupus nephritis via modulating anti-dsDNA antibody production and the inflammation followed IC deposition, and SAP-based intervening strategy may provide new approaches for treating SLE disease.

## Introduction

Defect in clearance of self nuclear antigen is the hallmark of systemic lupus erythematosus (SLE), an autoantibody-mediated chronic autoimmune disease characterized by the deposition of immune complexes and its followed inflammation that contribute to sever organ damage [1]–[3]. However, the precise means by which clearance of self antigen is inefficient in SLE remain obscure. Studies of both mice and humans suggest that SLE could arise from excessive production of self antigen released from unremoved apoptotic cells and impairment in the ability of macrophages to clear self antigen [4], [5].

Our previous study revealed that the syngeneic female BALB/c mice immunized with activated lymphocyte-derived DNA (ALD-DNA) develop high titers of anti-dsDNA antibodies, immune complex (IC) deposition, proteinuria, and glomerular nephritis which closely resemble human SLE [6]–[8], thus being used as a model to investigate pathogenesis and potential new therapies for human disease. These findings indicate that ALD-DNA, which mimics large amount of self-DNA released from unremoved apoptotic lymphocytes in SLE patients, might serve as an important self-immunogen to trigger the autoimmune responses which eventually lead to the pathogenesis of SLE in the murine model.

In addition to DNA overload in SLE, insufficiency of DNA clearance represents the other side of the coin [4]. Emerging studies reveal that serum amyloid P component (SAP) would be one of the candidates responsible for DNA clearance [2], [4]. SAP is a member of the pentraxin family of proteins and an acute phase reactant, which is produced primarily in the liver in response to infection, inflammation, and trauma [9]. SAP could recognize DNA and other ligands, activate complement, and facilitate pathogen and nuclear antigen phagocytosis, hence playing a nonredundant role in protection against autoimmune disease and in resistance against selected pathogens [10]. Furthermore, SAP shares many properties in common with IgG, including the capacity to interact with FcγR and the ability to bind to ligands [11], [12]. The interaction of SAP with FcγR mediates several functions that are analogous or opposite to those of IgG, including modulation the response to inflammatory stimuli and opsonization of bacteria and altered or exposed self-molecules on damages cells [4], [11], thus could compete with antibody and be used to treat antibody-mediated disease such as SLE.

As the major DNA- and chromatin-binding protein in plasma of mice, SAP could bind to nuclear antigens that are the target of the autoantibodies of patients with SLE, as well as to damaged membranes and microbial antigens [13], [14]. Furthermore, SAP−/− mice spontaneously developed antinuclear autoimmunity and sever glomerulonephritis, a phenotype resembling human SLE [15], which strongly supported a role for SAP in the protection against self-DNA and chromatin-induced autoimmunity.

Although the pathological relevance of SAP to autoimmune disease and the significance of self-DNA in the pathogenesis of SLE attracted much attention in recent years, whether SAP takes responsibility for self-DNA clearance and plays a protective role in self-DNA-induced SLE in a mouse model with clear genetic background remain poorly understood. In the present study, we tested the ratios of SAP to DNA and found that they decreased in ALD-DNA-induced lupus mice as compared to controls, and were negatively correlated with SLE disease. Further SAP gene administration in the early stage of SLE disease could reversed lupus nephritis via reduced pathogenic anti-dsDNA antibody production, while in the late stage of disease, SAP gene treatment alleviated proteuria and lupus nephritis via reducing the infiltration of leukocytes and the production of inflammatory markers besides decreasing the levels of anti-dsDNA antibodies. These results indicated that SAP administration would ameliorate self-DNA-induced lupus nephritis via regulating pathogenic anti-dsDNA antibody production and inflammation in lupus mice, which might provide SAP as a potential therapeutic strategy for self-antigen induced SLE and other autoimmune disease.

## Results

### ALD-DNA immunization induces SLE syndrome in non-autoimmune-prone mice

**Figure 1:**
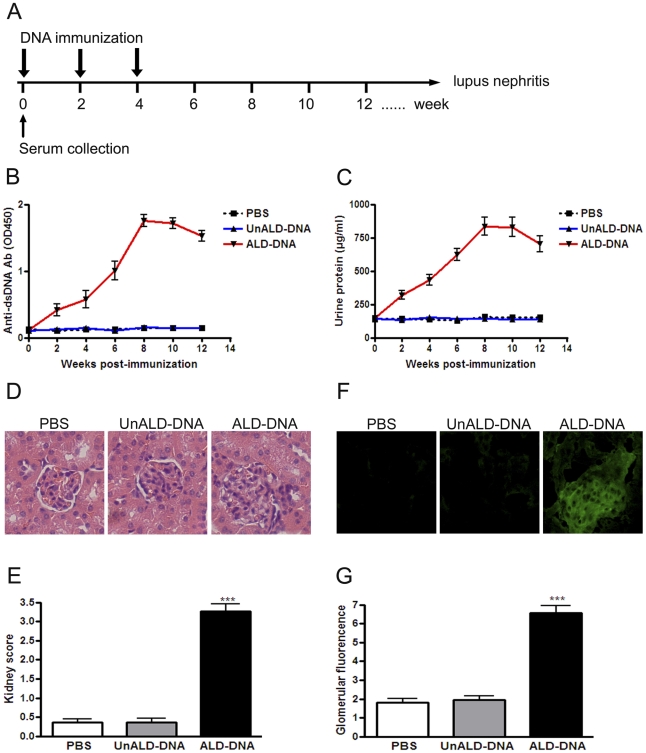
ALD-DNA immunization induces high levels of anti-dsDNA antibody and lupus nephritis. (A) Schematic diagram of animal immunization. 6- to 8-week old female BALB/c mice were immunized subcutaneously with ALD-DNA (50 µg/mice) plus CFA at week 0, followed by two booster immunizations of ALD-DNA (50 µg/mice) emulsified with IFA at week 2 and week 4 after initial immunization. (B) Serum anti-dsDNA IgG levels were measured by ELISA every 2 weeks after initial immunization. Data are means ± SD from 10 mice in each group. (C) Urine protein levels of the mice were assessed by BCA Protein Assay Kit every 2 weeks. Data are means ± SD from 10 mice in each group. (D) 8 weeks after initial immunization, nephritic pathology was evaluated by H&E staining of renal tissues. Imagines (magnification×200) are representative of at least 10 mice in each group. (E) The kidney score was assessed using paraffin sections stained with H&E in (D). ***, *p*<0.001. (F) Glomerular immune deposition were detected by direct immunofluorescence for IgG in frozen kidney section from ALD-DNA-immunized lupus mice or control mice. Representative images (magnification×200) of 10 mice are shown for each group. (G) Mean glomerular fluorescence intensity (arbitrary units) was determined for IgG in ALD-DNA-immunized lupus mice (n = 10) and control mice (n = 10). ***, *p*<0.001.

Levels of serum anti-dsDNA antibodies, which represent a serological hallmark of SLE, tend to reflect disease severity for SLE patients [1]. According to our previously reported procedure, SLE murine model was generated by immunizing female BALB/c mice with ALD-DNA (Fig. 1A) [6], [7]. Compared with PBS or unactivated lymphocyte-derived DNA (UnALD-DNA) injection, ALD-DNA immunization generated higher levels of anti-dsDNA IgG antibody (Fig. 1B), which was evident from week 4 and reached the maximum at week 8 after initial injection (Fig. 1B). Glomerulonephritis was also confirmed by urine protein quantification, H&E staining of renal tissues, and immune complex deposition assay (Fig. 1C–G). Remarkably up-regulated urine protein (Fig. 1C), notable glomerulonephritis (Fig. 1D and E), and increased IgG deposition (Fig. 1F and G) were found in ALD-DNA-immunized lupus mice as compared to PBS- or UnALD-DNA-treated controls (Fig. 1C–G). These results demonstrate that SLE murine model could be established through ALD-DNA immunization.

### The ratios of SAP to DNA decrease in lupus mice and are negatively correlated with SLE disease

**Figure 2:**
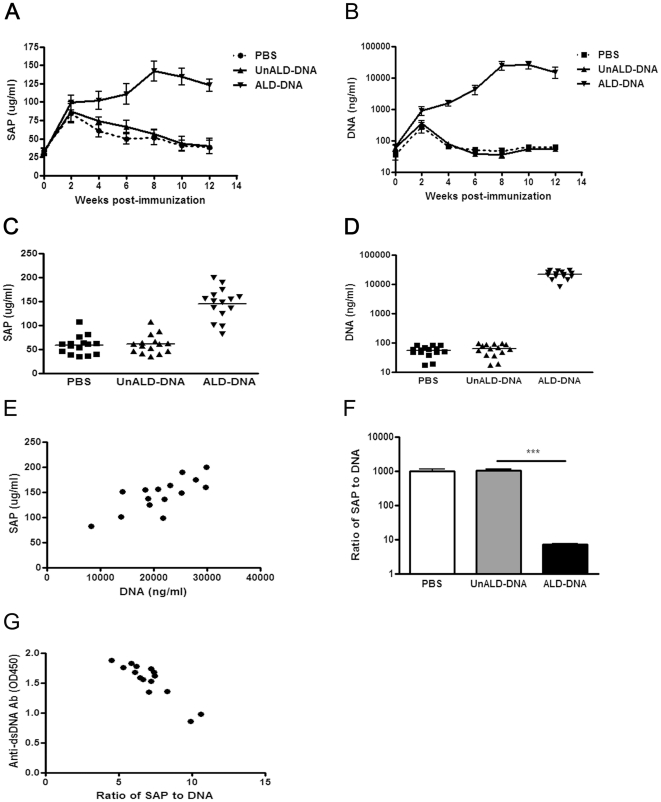
The ratios of SAP to DNA decrease in SLE murine model. 6-week-old female BALB/c mice were immunized subcutaneously with ALD-DNA, UnALD-DNA, or PBS (n = 15) for 3 times in 4 weeks. (A) The dynamics of SAP level in serum of mice immunized with ALD-DNA, UnALD-DNA, or PBS were determined by ELISA assay every 2 weeks. (B) The dynamics of circulating DNA level in serum of mice immunized with ALD-DNA, UnALD-DNA, or PBS were determined using a PicoGreen DNA detection kit (Invitrogen) every 2 weeks. (C) SAP levels in the serum of SLE murine model and controls were tested by ELISA at week 8 after initial immunization. n = 15. (D) Circulating DNA levels in the serum of SLE murine model and controls were determined using a PicoGreen DNA detection kit at week 8 after initial immunization. n = 15. (E) The correlation between SAP and DNA levels in ALD-DNA-immunized lupus mice. n = 15. Pearson correlation analysis was used to carry out the correlation study. r = 0.76; *P*<0.05. (F) The ratios of SAP to DNA in SLE murine model and controls. Data are means ± SD from 15 mice in each group. ***, *P*<0.001. (G) The correlation between the ratio of SAP to DNA and anti-dsDNA IgG level in SLE murine model. Pearson correlation analysis was used to carry out the correlation study. r = 0.90; *P*<0.001.

To study whether SAP has a correlation to SLE disease, the levels of SAP and circulating DNA in the serum of lupus mice generated by ALD-DNA immunization were assayed. Slightly increased serum SAP levels accompanied with remarkably enhanced circulating DNA levels were found in lupus mice as compared with those in controls (Fig. 2A–D). Pearson correlation analysis showed that the serum SAP levels were closely correlated to the circulating DNA levels (Fig. 2E). However, the ratios of SAP to DNA were lower in lupus mice than in controls, which suggested that SAP protein were relatively insufficient in lupus mice (Fig. 2F). Notably, the ratios of SAP to DNA were negatively correlated with the levels of anti-dsDNA antibodies in SLE mice (Fig. 2G). Taken together, these results indicate that SAP was relatively insufficient in lupus mice.

### Efficiently expressed SAP protein could inhibit ALD-DNA-mediated innate immune responses *in vitro*

**Figure 3:**
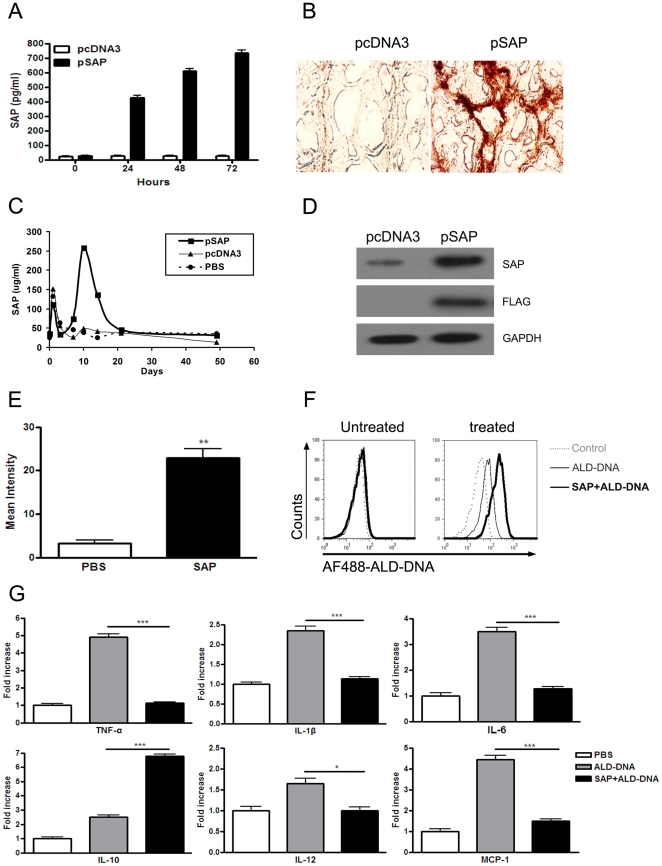
Efficiently expressed SAP could inhibit ALD-DNA-induced innate immune response. (A) NIH 3T3 cells were transfected with pcDNA3 or pcDNA3-SAP plasmid (pSAP) using Lipofectamine 2000 transfection reagent, and cell supernatants were collected and subjected to ELISA assay for the expression of SAP protein at 24 h, 48 h, and 72 h post transfection. Data are means ± SD of three independent experiments. (B–D) 6-week-old female BALB/c mice were injected intramuscularly with pSAP (100 µg/mice). (B) Immunohistochemistry examination was performed to determine the expression of SAP protein with anti-mouse SAP Ab in the muscle received pSAP injection. Imagines (×400) are representative of at least 10 mice in each group. (C) ELISA assay was performed to determine the levels of SAP protein in the serum of mice received pSAP injection. Data are means ± SD of three independent experiments. n = 10. (D) The serum was collected on day 10 after pSAP injection. Western blot analysis was performed to detect the expression of SAP in serum of mice received pSAP injection. Data are representative of at least 10 mice in each group. (E–G) SAP protein was purified from the serum of mice received pSAP injection. (E) The binding ability of the purified mouse SAP protein to DNA was detected by dot blot analysis. Quantitative analysis of blots was reflected as mean intensity. Data are means ± SD of three independent experiments. **, *P*<0.01. (F) Alexa Fluor 488 labeled ALD-DNA (AF488-ALD-DNA) was incubated with or without the purified SAP protein for 2 h (SAP plus ALD-DNA). BMDMs were treated with or without chloroquine (100 µg/ml) before DNA incubation. The intracellular Alexa Fluor 488 labeled ALD-DNA (AF488-ALD-DNA) in chloroquine-treated or untreated macrophages was determined by flow cytometry. Data are representative of results obtained in three independent experiments. (G) The purified SAP protein was incubated with ALD-DNA (SAP plus ALD-DNA) for 2 h. RAW264.7 cells were treated with PBS, ALD-DNA, or SAP plus ALD-DNA. 12 h later, levels of TNF-α, IL-1β, IL-6, IL-10, IL-12, and MCP-1 in the RAW264.7 cells were measured by real-time PCR. Data are means ± SD of three independent experiments. * *P*<0.05; ***, *P*<0.001.

Our results described above provided the basis for the hypothesis that SAP administration *in vivo* may modulate the immune response in SLE disease. Consequently, pcDNA3-SAP recombinant (pSAP) was constructed for expression of SAP. As shown in Fig. 3A, ELISA analysis for the expression of SAP in culture supernatants of NIH3T3 cell line transfected with pSAP shown that SAP cDNA cloned into pcDNA3 could be correctly transcripted, translated and the protein could be efficiently secreted. To detect the expression of SAP *in vivo*, BALB/c mice were injected intramuscularly with pSAP (100 µg/mice). Immunohistochemistry examination showed an obvious expression of SAP in the muscle received pSAP compared with that receiving pcDNA3 (Fig. 3B). Consistently, quantitative analysis of SAP levels in serum of mice revealed that SAP reached maximal levels at day 10 after injection and then declined (Fig. 3C). 21 days after plasmid injection, the concentration of serum SAP protein went back to the baseline level (Fig. 3C). In contrast, the levels of serum SAP protein in the mice treated with parental plasmid pcDNA3 or physiological saline were not significantly increased over the course of the experiments (Fig. 3C). To further confirm the expression of SAP *in vivo*, serum was collected on day10 after pSAP injection and subjected to western blot analysis using specific anti-SAP antibody. Marked immune-reactive bands were observed in the serum from mice receiving pSAP injection (Fig. 3D), indicating that SAP could be efficiently expressed *in vivo*. Accumulating data indicate that SAP has the capacity of binding to DNA under physiological conditions [9]. To explore the biological function of SAP purified from the serum of mice receiving pSAP injection, the binding ability of the purified SAP protein to ALD-DNA was evaluated. It was found that the purified SAP protein had the capacity to bind to ALD-DNA (Fig. 3E). Previous studies have shown that SAP can bind macrophage and opsonize the ligands for phagocytosis [11], [16]. In order to investigate whether the binding of SAP to ALD-DNA had any effects on the uptake of ALD-DNA by macrophages, we performed flow cytometry to determine the uptake of ALD-DNA or the complexes of purified SAP protein and ALD-DNA (SAP plus ALD-DNA). However, the intracellular DNA did not increase in macrophages in the presence of SAP (Fig. 3F). As endocytic naked DNA was always degraded rapidly by endosomal nucleases, we used chloroquine to prevent endosome acidification. It was found that SAP increased the intracellular fluorescence rates of treated macrophages, indicating that SAP binding to ALD-DNA promoted the uptake of ALD-DNA by macrophages (Fig. 3F). Furthermore, we performed real-time PCR to detect cytokine expression in the macrophages cultured with ALD-DNA or the complexes of purified SAP protein and ALD-DNA (SAP plus ALD-DNA), and found that mRNA levels of inflammatory cytokines including TNF-α, IL-1β, IL-6, IL-12, and MCP-1 were notably decreased in the macrophages cultured with SAP plus ALD-DNA; however, mRNA level of IL-10 was significantly increased in the macrophages cultured with SAP plus ALD-DNA as compared with those of macrophages cultured with ALD-DNA alone (Fig. 3G). These data suggest that pSAP plasmid could be correctly transcripted, translated and the expressed SAP protein could inhibit ALD-DNA-mediated innate immune responses *in vitro*.

### pSAP treatment in the early stage of SLE disease reverses lupus nephritis via reducing anti-dsDNA antibody production and IC deposition

**Figure 4:**
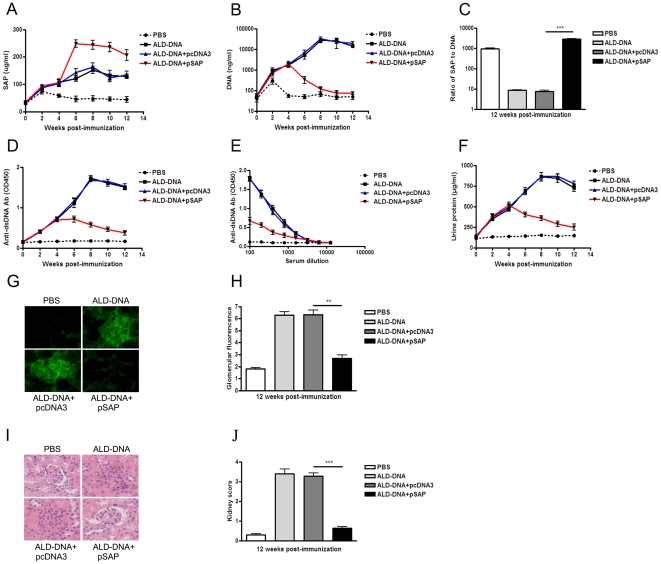
pSAP administration in the early stage of SLE disease reverses ALD-DNA-induced lupus nephritis. BALB/c mice were immunized subcutaneously with ALD-DNA (50 µg/mouse) or PBS for total 3 times in 4 weeks. Mice immunized with ALD-DNA were administrated intramuscularly with pSAP (100 µg/mice) from week 4 after initial immunization (with the onset of proteinuria) and injected every 2 weeks for total 5 times. (A) The dynamics of SAP level in serum of lupus mice injected with pSAP (ALD-DNA plus pSAP) or pcDNA3 (ALD-DNA plus pcDNA3) were determined by ELISA assay every 2 weeks. (B) The dynamics of circulating DNA level in serum of lupus mice injected with pSAP (ALD-DNA plus pSAP) or pcDNA3 (ALD-DNA plus pcDNA3) were determined by ELISA assay every 2 weeks. (C) The ratios of SAP to DNA in SLE murine model injected with pSAP (ALD-DNA plus pSAP) or pcDNA3 (ALD-DNA plus pcDNA3) at week 12 after initial immunization. Data are means ± SD from 10 mice in each group. ***, *P*<0.001. (D) Serum anti-dsDNA IgG levels of the mice were measured by ELISA assay every 2 weeks. (E) Anti-dsDNA IgG antibody titers in serum of pSAP-treated lupus mice (ALD-DNA plus pSAP) or pcDNA3-treated lupus mice (ALD-DNA plus pcDNA3) were detected by ELISA assay at week 8 after the initial ALD-DNA immunization. n = 10. (F) Urine protein levels of the mice were assessed by BCA Protein Assay Kit (Thermo Fisher Scientific) every 2 weeks. n = 10. (G) The deposition of IgG-containing IC in glomeruli at week 12 after initial immunization. Imagines (×200) are representative of at least 10 mice in each group. (H) Mean glomerular fluorescence intensity (arbitrary units) was determined for IgG in ALD-DNA-immunized lupus mice and control mice at week 12 after initial immunization. n = 10. **, *P*<0.01. (I) 12 weeks after initial immunization, nephritic pathological changes were shown by H&E staining of renal tissues surgical resected from the mice. Imagines (×200) are representative of at least 10 mice in each group. (J) The kidney score was assessed using paraffin sections stained with H&E. n = 10. ***, *P*<0.001.

To evaluate the effect of pSAP treatment in mice, ALD-DNA-induced lupus mice with the onset of proteinuria (at week 4 after the initial ALD-DNA immunization) were treated with pSAP (ALD-DNA plus pSAP group). Significantly increased serum SAP levels accompanied with remarkably decreased circulating DNA levels were found in pSAP-treated lupus mice as compared with those in pcDNA3-treated lupus mice (Fig. 4A and B). The ratios of SAP to DNA were simultaneously increased in pSAP-treated lupus mice as compared with those in pcDNA3-treated lupus mice, which suggested that pSAP injection could reverse the insufficiency of SAP in lupus mice (Fig. 4C). 12 weeks after the initial ALD-DNA immunization, the levels of anti-dsDNA autoantibodies, IC deposition, proteinuria, renal pathology, and kidney score were analyzed. Notably reduced the levels of anti-dsDNA autoantibodies (Fig. 4D and E), urine protein (Fig. 4F), IC deposition (Fig. 4G and H), renal pathology (Fig. 4I), and kidney score (Fig. 4J) were found in the pSAP-treated lupus mice as compared with those of pcDNA3-treated lupus mice. These results show that pSAP treatment in the early stage of SLE disease could reverse lupus nephritis via decreasing anti-dsDNA antibody production and IC deposition in lupus mice.

### pSAP administration in the late stage of SLE disease alleviates lupus nephritis via reducing leukocyte infiltration and inflammatory marker production

**Figure 5:**
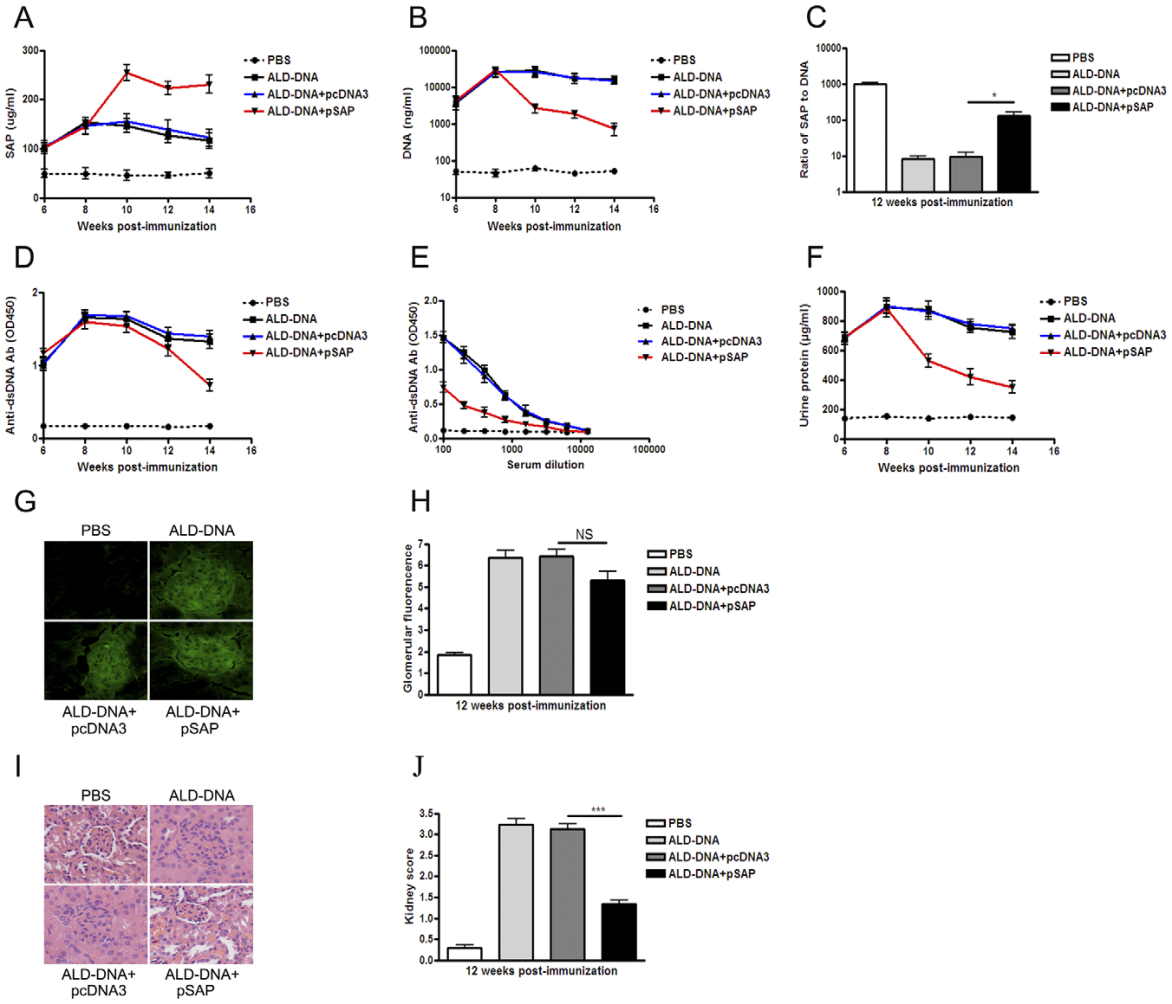
pSAP administration in the late stage of SLE disease ameliorates ALD-DNA-induced lupus nephritis. BALB/c mice were immunized subcutaneously with ALD-DNA (50 µg/mouse) or PBS for total 3 times in 4 weeks. Mice were administrated intramuscularly with pSAP (100 µg/mice) from week 8 after initial immunization (when sever lupus nephritis has been established) and injected every 2 weeks for total 4 times. (A) The dynamics of SAP level in serum of lupus mice injected with pSAP (ALD-DNA plus pSAP) or pcDNA3 (ALD-DNA plus pcDNA3) were determined by ELISA assay every 2 weeks. (B) The dynamics of circulating DNA level in serum of lupus mice injected with pSAP (ALD-DNA plus pSAP) or pcDNA3 (ALD-DNA plus pcDNA3) were determined by ELISA assay every 2 weeks. (C) The ratios of SAP to DNA in SLE murine model injected with pSAP (ALD-DNA plus pSAP) or pcDNA3 (ALD-DNA plus pcDNA3) at week 12 after initial immunization. Data are means ± SD from 10 mice in each group. ***, *P*<0.001. (D) Serum anti-dsDNA IgG levels were measured by ELISA assay every 2 weeks. (E) Anti-dsDNA IgG antibody titers in serum of pSAP-treated lupus mice (ALD-DNA plus pSAP) or pcDNA3-treated lupus mice (ALD-DNA plus pcDNA3) were detected by ELISA assay at week 14 after the initial ALD-DNA immunization. n = 10. (F) Urine protein levels of the mice were assessed by BCA Protein Assay Kit (Thermo Fisher Scientific) every 2 weeks. n = 10. (G) The deposition of IgG-containing IC in glomeruli at week 12 after initial immunization. Imagines (×200) are representative of at least 10 mice in each group. (H) Mean glomerular fluorescence intensity (arbitrary units) was determined for IgG in ALD-DNA-immunized lupus mice and control mice at week 12 after initial immunization. n = 10. NS, not significant. (I) 12 weeks after initial immunization, nephritic pathological changes were shown by H&E staining of renal tissues surgical resected from the mice. Imagines (×200) are representative of at least 10 mice in each group. (J) The kidney score was assessed using paraffin sections stained with H&E. n = 10. ***, *P*<0.001.

To further evaluate the protective effect of pSAP treatment in mice in the late stage of SLE disease, ALD-DNA-induced lupus mice with the established lupus nephritis (at week 8 after the initial ALD-DNA immunization) were treated with pSAP (ALD-DNA plus pSAP group). Significantly increased serum SAP levels accompanied with remarkably decreased circulating DNA levels were found in pSAP-treated lupus mice as compared with those in pcDNA3-treated lupus mice (Fig. 5A and B). The ratios of SAP to DNA were simultaneously increased in pSAP-treated lupus mice as compared with those in pcDNA3-treated lupus mice, which suggested that pSAP injection could partly improve the insufficiency of SAP in lupus mice (Fig. 5C). 12 weeks after the initial ALD-DNA immunization, the levels of anti-dsDNA autoantibodies, IC deposition, proteinuria, renal pathology, and kidney score were analyzed in the lupus murine model receiving pSAP injection at week 8 after the initial ALD-DNA immunization when lupus mice already had the highest levels of anti-dsDNA autoantibodies and established lupus nephritis (ALD-DNA plus pSAP group). Twelve weeks after the initial immunization, notably decreased levels of urine protein (Fig. 5F) and ameliorated glomerulonephritis (Fig. 5I and J) but slowly reduced levels of autoantibody titers (Fig. 5D and E) and IC deposition (Fig. 5G and H) were found in pSAP-treated lupus mice as compared with pcDNA3-treated lupus mice, indicating the improved lupus nephritis was not exclusively ascribed to the decreased anti-dsDNA antibody levels and IC deposition.

**Figure 6:**
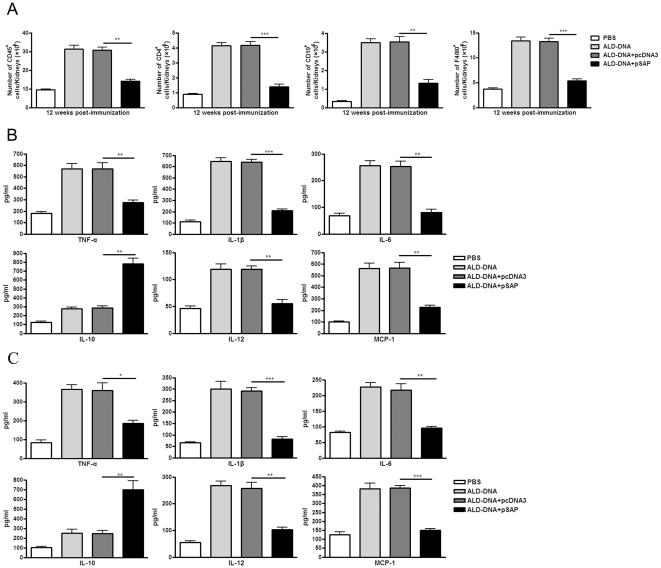
pSAP administration in the late stage of SLE disease reduces inflammation in kidneys of mice. BALB/c mice were immunized subcutaneously with ALD-DNA (50 µg/mouse) for total 3 times in 4 weeks. Mice were administrated intramuscularly with pSAP (100 µg/mice) from week 8 after initial immunization and injected every 2 weeks for total 4 times. (A) 12 weeks after initial immunization, the infiltration of leukocyte populations in kidneys of treated mice were assessed by flow cytometry. Decreased CD45^+^ cells, CD19^+^ cells, CD4^+^ cells, and F4/80^+^ cells were found in kidneys of pSAP-treated lupus mice. Data are representative of results obtained in three independent experiments. n = 10. (B) At week 12, the kidney tissues were collected and homogenized, the expression of TNF-α, IL-1β, IL-6, IL-10, IL-12, and MCP-1 were determined by ELISA assay. Data are means ± SD of three independent experiments. n = 8. ** *P*<0.01, *** *P*<0.001. (C) At week 12 after initial immunization, expression of TNF-α, IL-1β, IL-6, IL-10, IL-12, and MCP-1 in serum of the mice were determined by ELISA assay. Data are means ± SD of three independent experiments. n = 8. * *P*<0.05, ** *P*<0.01, *** *P*<0.001.

Other than the pathogenic anti-dsDNA autoantibody production and IC deposition, severe renal injury can be mediated by infiltrating proinflammatory leukocyte populations [17]. Flow cytometry analysis of cells extracted from kidneys of lupus mice showed a marked decrease in the number of CD45^+^ leukocytes in kidneys isolated from pSAP-treated lupus mice as compared with pcDNA3-treated lupus mice (Fig. 6A). Further flow cytometry analysis of infiltrating leukocyte populations revealed that pSAP-treated lupus mice displayed a decrease in renal T cells (CD4^+^) and B cells (CD19^+^) as compared to pcDNA3 treated lupus mice (Fig. 6A), but T and B cells only account for a portion of the decreased infiltrating cells in the kidneys of pSAP-treated lupus mice. Flow cytometry analysis of the presence of myeloid cells showed that pSAP-treated lupus mice exhibited a notable decrease in the number of F4/80^+^ macrophages (Fig. 6A), but there was no significant decrease in the number of CD11c^+^ dendritic cells (data not shown), suggesting that macrophages were the key cells that was influenced by pSAP treatment. As a set of inflammatory markers mainly secreted by macrophages were expressed in kidneys following glomerular immune complex deposition [8], [18], [19], further studies using ELISA analysis allowed us to determine several key markers in kidneys of mice. It was found that TNF-α, IL-1β, IL-6, IL-12 and MCP-1, which were upregulated in kidneys of ALD-DNA-induced lupus mice, were decreased in the pSAP-treated lupus mice (Fig. 6B). However, levels of IL-10 were notably increased in the pSAP-treated lupus mice (Fig. 6B). Analysis of the cytokine profile in serum of mice further confirmed that the inflammatory markers (including TNF-α, IL-1β, IL-6, IL-12, and MCP-1) were extensively and dramatically decreased in pSAP-treated lupus mice as compared with other control groups (Fig. 6C). These data suggest that pSAP treatment in the late stage of SLE disease could ameliorate the lupus nephritis via reducing the number of infiltrating inflammatory cells and decreasing the levels of inflammatory markers.

## Discussion

We have provided here evidence that relative insufficiency of SAP played a critical role in the pathological process of the ALD-DNA-induced lupus nephritis, and verified that administration of SAP *in vivo* by a plasmid encoding the SAP, could significantly ameliorated the severity of SLE disease, as demonstrated by decreased levels of anti-dsDNA antibodies, reduced immune complex deposition, less proteinuria, less lupus nephritis, and decreased kidney score of glomerulonephritis. This therapeutic effect was closely associated with reduced production of anti-dsDNA antibodies in the early stage of the disease and significantly decreased infiltrating lymphocytes and reduced levels of inflammatory markers in kidneys of pSAP-treated mice in the late stage of the disease.

In previous study, the crucial and versatile functions of SAP in autoimmune disease have been well established [9], [10]. SAP^−/−^ mice spontaneously develop antinuclear autoimmunity and severe glomerulonephritis, a phenotype resembling human SLE [15]. However, people doubt if SAP deficiency or strain combination contributes to the pathogenesis of SLE [20], [21]. And the SAP-linked genes co-deficency may confuse the elucidation of the role of SAP in autoimmunity [22]. Therefore, study of SLE pathogenesis in regarding to SAP in a mouse model with clear genetic background is very critical and should be a prerequisite. Herein, we use ALD-DNA-induced SLE murine model to extensively study the role of SAP in SLE pathogenesis. In this study, it was found that the ratios of SAP to DNA significantly decreased in ALD-DNA-induced lupus mice as compared to controls. SAP plasmid (pSAP) treatment *in vivo* could significantly increase the levels of serum SAP and notably decreased the levels of circulating DNA, thus simultaneously increasing the ratios of SAP to DNA. These results indicated that SAP was relative insufficient in ALD-DNA-induced SLE mice, which further provide the evidence that SAP defect rather than the deficiency of SAP linked genes might contribute to the pathogenesis of antinuclear autoimmunity in SAP^−/−^ mice [15], [20]–[22]. Notably, the ratios of SAP to DNA were negatively correlated with the titers of anti-dsDNA antibodies in lupus mice, which verified the critical role of SAP insufficiency in ALD-DNA-induced autoimmunity, although we did not exclude other factors contributing to the pathogenesis of the SLE disease [23], [24]. As SAP and IgG shared the same binding site on FcγR and competed for FcγR binding, SAP could be used to inhibit antibody or immune complex-mediated immune response [11]. All these results strongly support a role for SAP in the protection against self-DNA-induced autoimmunity.

We thus adopted a gene therapy method using the pcDNA3-SAP plasmid (pSAP) to treat lupus nephritis. The SAP protein could be efficiently expressed and secreted into the culture supernatants when pSAP was transfected into NIH3T3 cell line, indicating that SAP cDNA cloned into pcDNA3 could be correctly transcripted, translated and the protein was efficiently secreted *in vitro*. Further study confirmed that the SAP protein could be efficiently expressed and secreted into the systemic circulation via intramuscular injection of pSAP, and SAP protein purified from pSAP-treated mice could promote self-DNA clearance via binding to self-DNA, indicating that efficiently expressed SAP protein could perform the biological functions. Analysis of cytokine levels of macrophages cultured with the complexes of SAP and DNA revealed that levels of inflammatory cytokines including TNF-α, IL-1β, IL-6, IL-12, and MCP-1 were notably decreased in the macrophages cultured with SAP plus ALD-DNA; however, level of IL-10 was significantly increased in the macrophages cultured with SAP plus ALD-DNA as compared with those of macrophages cultured with ALD-DNA alone, indicating that SAP inhibited ALD-DNA-mediated innate immune response *in vitro*. However, the mechanism of how SAP promotes the clearance of ALD-DNA needs to be further revealed. The protective effect of SAP had also been noticed by other groups and testified that the interaction of SAP with FcγR is able to mediate phagocytosis of apoptotic cells, as well as mediate protective immune response [12], [25]. In our study, injection of pSAP in the early stage of SLE disease with the onset of proteinuria notably reversed lupus nephritis. The beneficial effect of pSAP treatment was associated with its inhibitory effect on ALD-DNA-induced anti-dsDNA antibody production and immune complex deposition, which was consistent with the findings from other groups that anti-dsDNA antibodies promoted initiation of lupus nephritis [26]. Furthermore, injection of pSAP at the late stage of SLE disease with the established lupus nephritis remarkably reduced proteinuria and lupus nephritis, while slowly decreased levels of anti-dsDNA antibodies and immune complex deposition were noticed in these pSAP-treated mice, indicating that the protective effect of SAP treatment in the late stage of SLE disease was not exclusively attributable to the decreased levels of anti-dsDNA autoantibodies.

SAP is the main acute-phase reactants in mice and its capacity to bind with DNA has been identified for more than 20 years [13], [14], [27], [28]. However, recent emerging evidences indicate that SAP also plays a critical role in modulating cytokine production in inflammatory reactions [10], [11]. The inflamed kidneys of patients with SLE, NZB/W F_1_ mice, and ALD-DNA-induced lupus mice contain many lymphocytes around glomeruli, blood vessels, and in the interstitium [8], [29]–[31]. Our study verified again that CD45^+^ leucocytes were infiltrated into kidneys of ALD-DNA-induced lupus mice. SAP treatment in the late stage of SLE disease significantly decreased the numbers of CD45^+^ leucocytes including T cells, B cells, and macrophages but not dendritic cells in kidney tissue of ALD-DNA-induced lupus mice and the underlying mechanisms need to be elucidated in the future. Further analysis of inflammatory markers revealed that SAP treatment notably decreased systemic and local inflammatory cytokine levels of TNF-α, IL-1β, IL-6, IL-12, and MCP-1, which were closely associated with the severity of lupus nephritis [18]. However, inhibitory cytokine IL-10 was notably increased in pSAP-treated lupus mice, which might also partly contribute to the alleviation of lupus nephritis. Our results were in good agreement with previous studies, and further confirmed the immunomodulatory function and potential protective and therapeutic effect of SAP in ALD-DNA-induced lupus nephritis.

In conclusion, we showed that SAP gene administration at the onset of proteinuria could reverse lupus nephritis. The main mechanism seems to be associated with the effective inhibiting the production of anti-dsDNA antibodies and immune complex deposition. While SAP gene treatment in the stage of established lupus nephritis could reduce the disease severity, which was possibly attributable to the decreased infiltration of lymphocytes and reduced levels of inflammatory markers beyond decreased anti-dsDNA autoantibody production. Our findings may provide an insight into better understanding of the underlying mechanism of ALD-DNA-induced lupus nephritis, and provide the preclinical data indicating that SAP administration can alleviate lupus nephritis. This strategy may be a clinically relevant and feasible therapeutic method for patients suffering from self-DNA-induced nephritis or other autoimmune diseases which accompany with decreased clearance of apoptotic cells.

## Materials and Methods

### Ethics statement

All experiments carried out in this study were strictly performed in a manner to minimize suffering of laboratory mice. All animal procedures were performed according to the Guide for the Care and Use of Medical Laboratory Animals (Ministry of Health, P.R. China, 1998) and with the ethical approval of the Shanghai Medical Laboratory Animal Care and Use Committee (Permit number: SYXK 2007-0036) as well as the Ethical Committee of Fudan University (Permit number: 2007016).

### Mice and plasmid

Six-week-old female BALB/c mice were purchased from the Experimental Animal Center of Chinese Academy of Sciences (Shanghai, P. R. China). Mice were housed in a specific pathogen-free room under controlled temperature and humidity. The full length of SAP cDNA was amplified from total RNA of murine liver using the primers 5′-CGA AGC TTG CCA CCA TGG ACA AGC TGC TGC-3′ and 5′-CGG AAT TCC CTC TTA CAC ATC GGC AAT C-3′. 24 nucleotides encoding FLAG epitope (DYKDDDDK) were added directly at the carboxyl-terminal of SAP gene sequence by primer design. SAP cDNA with a FLAG tag was inserted into the pcDNA3 vector (Invitrogen) to generate pcDNA3-SAP plasmid (pSAP). The plasmid construct was confirmed by DNA sequencing.

### Generation of bone marrow-derived macrophages (BMDMs)

Bone marrow (BM) cells were harvested from uninfected, normal BALB/c mice and filtered through nylon mesh. BM cells were cultured in L929 cell-conditioned medium at a density of 3×10^5^ cells/ml of medium and maintained in a 5% CO_2_ incubator at 37°C as described previously [32], [33]. Six days after initial BM cells culture, the medium was changed and the purity of F4/80^+^ cells was more than 90%, as determined by flow cytometry (FACSCalibur; BD Biosciences).

### DNA preparation

ALD-DNA and UnALD-DNA were prepared with murine splenocytes which were generated from surgical resected spleens of six- to eight-week-old female BALB/c mice and cultured with or without Con A (Sigma-Aldrich) *in vitro* as previously described [7]. Briefly, for generation of ALD-DNA, splenocytes were seeded at 2×10^6^ cells/ml in 75 cm^2^ cell culture flask and cultured in the presence of Con A (5 µg/ml) for 6 days to induce apoptosis. The apoptotic cells were stained with FITC-labeled Annexin V (BD Biosciences) and propidium iodide (PI; Sigma-Aldrich), and sorted using a FACSAria (BD Biosciences). Genomic DNAs from syngeneic apoptotic splenocytes were treated with S1 nuclease (TaKaRa) and proteinase K (Sigma-Aldrich), and then purified using the DNeasy Blood & Tissue Kits (Qiagen) according to the manufacturer's instructions. UnALD-DNA was prepared with unactivated (resting) splenocytes and extracted using the same methods. To exclude contaminations with LPS, sterile endotoxin-free plastic ware and reagents were used for DNA preparation. DNA samples were also monitored for low level of endotoxin by the Limulus amoebocyte lysate assay (BioWhittaker) according to the manufacturer's instructions. The concentration of DNA was determined by detection of the absorbance (A) at 260 nm. The apoptotic DNA ladder of ALD-DNA was confirmed by agarose gel electrophoresis (AGE).

### Generation of SLE murine model

To generate SLE murine model, 6- to 8-wk-old syngeneic female BALB/c mice were divided into several groups of 8–10 mice and actively immunized by subcutaneous injection on the back with 0.2 ml of an emulsion containing ALD-DNA (50 µg/mouse) in phosphate-buffered saline (PBS) plus equal volume of complete Freund's adjuvant (CFA; Sigma-Aldrich) at week 0, and followed by two booster immunizations of ALD-DNA (50 µg/mouse) emulsified with IFA (Sigma-Aldrich) at week 2 and week 4 for total 3 times as previously described [6], [7]. Eight to 10 mice in each group received an equal volume of PBS plus CFA or IFA, or UnALD-DNA (50 µg/mouse) plus CFA or IFA were used as contols. Mice were bled from retro-orbital sinus prior to immunization and at 2-week internals until 3 months after the initial immunization. 8 or 12 weeks later, mice were sacrificed and surgical resected spleens and kidneys were collected for further cellular function and tissue histology analysis.

### Autoantibody and proteinuria examination

Anti-dsDNA antibodies in the mice serum were determined by ELISA assay as described previously [6]. In briefly, ELISA plates (Costar) were pretreated with protamine sulphate (Sigma-Aldrich) and then coated with calf thymus dsDNA (Sigma-Aldrich). After incubation with mouse serum, the levels of anti-dsDNA Abs were detected with the horseradish peroxidase (HRP)-conjugated goat anti-mouse IgG (Southern Biotech). Tetramethylbenzidine (TMB) substrate was used to develop colors and absorbance at 450 nm was measured on a microplate reader (BIO-TEK ELX800). Proteinuria of the mice was measured with the BCA Protein Assay Kit (Thermo Scientific) according to the manufacturer's instructions.

### Measurement of anti-dsDNA antibody titers

Anti-dsDNA antibody titers in the mice serum were determined by ELISA assay as described previously [34]. In briefly, protamine sulphate pre-treated 96-well microtitre plates (Costar) were coated with calf thymus dsDNA (Sigma-Aldrich; 50 µg/ml) for 2 h at 37°C and then placed overnight at 4°C. After washing three times with PBS containing 0.05% Tween-20 (PBST), the plates were blocked with 1% BSA for 1 h, and serial dilutions of serum in PBS–1% BSA were added for 1 h at 37°C. After washing, the plates were incubated with 1∶1000 dilution of horseradish peroxidase (HRP)-conjugated goat anti-mouse IgG (Southern Biotech) for 1 h at 37°C. Tetramethylbenzidine (TMB) substrate was used to develop colors and absorbance at 450 nm was measured on a microplate reader (BIO-TEK ELX800).

### Measurement of SAP level

To assess protein levels of SAP in serum of mice or in the culture supernatants, ELISA assays were performed with the following anti-SAP Abs and SAP standards: sheep anti-mouse SAP (Calbiochem), rabbit anti-mouse SAP (Calbiochem), and mouse SAP (Calbiochem) as previously described [35].

### Measurement of circulating DNA level

DNA was extracted from serum samples and then quantified using a PicoGreen DNA detection kit (Invitrogen) according to the manufacturer's instructions [36]. In briefly, DNA was extracted from 200 µl of serum samples using a QIAamp Blood Kit (Qiagene) using the blood and body fluid protocol as recommended by the manufacturer. After the removal of most proteins by digestion with proteinase K, the sample was applied to the QIAamp 96 plate. DNA was adsorbed onto the silica membrane during a brief centrifugation step, while any remaining protein, salt and other contaminants were completely removed by three consecutive washes. Membrane-bound DNA was then eluted in double deionized H_2_O or Tris–EDTA buffer. A final elution volume of 200 µl was used. Quantification of DNA was carried out using a PicoGreen DNA detection kit (Invitrogen). Calf thymus DNA (100 mg/ml; Sigma-Aldrich) was used as the standard. The concentration of DNA in the standard curve ranged from 0 to 100 ng/ml. Briefly, 20 ml of final DNA eluated was mixed with 1 ml of Tris–EDTA (10 mmol/l Tris–HCl, 1 mmol/l EDTA, pH 7.5) diluted with PicoGreen reagent. Fluorescence intensity was measured on an F-2000 spectrofluorometer (Molecular Devices) at excitation wavelength of 480 nm and an emission of 520 nm. Standard curve used to determine the levels of circulating DNA in the samples was established by the linear relationship between the known concentrations of calf thymus DNA (Sigma-Aldrich) and the corresponding fluorescence intensities.

### Immunohistochemistry examination

The expression of SAP protein in the muscle tissue received pcDNA3-SAP (pSAP) injection was analyzed by immunohistochemistry. In briefly, mice were injected with pSAP (100 µg/mice) at the site of femoral muscle. 3 days later, the muscle tissue harvested from pSAP treated mice were fixed in 4% paraformaldehyde, processed on a standard histology processor, embedded in paraffin, and cut into 5 micron sections. Paraffin sections were dewaxed in xylene and rehydrated in decreasing concentrations of alcohol. Sections were exposed to citrate buffer and heat antigen retrieval and then blocked and incubated with rabbit anti-mouse SAP antibody (Calbiochem). Sections were subsequently assayed with the Super Sensitive Polymer-HRP IHC Detection System (Vector Laboratories) according to the manufacturer's instructions. 3, 3′-Diaminobenzidine (DAB) substrate (Dako) was used to develop slides. Slides were counterstained with hematoxylin (Dako) and coverslipped using Permount mounting media (Fisher Scientific). Pictures were acquired with a 20×/0.45 Plan Fluor object on a Nikon SCLIPSS TE2000-S microscope (Nikon) equipped with ACT-1 software (Nikon). Original magnification was 400×.

### Western blot analysis

Six-week-old female BALB/c mice were injected intramuscularly with pSAP (100 µg/mice). 10 days later, the serum was collected and western blot analysis was performed as described previously [37]. In briefly, serum was electrophoresed on SDS-PAGE gels and then transferred to the PVDF membrane. The membrane was probed with rabbit anti-SAP (Calbiochem), mouse anti-FLAG (Santa Cruz), or rabbit anti-GAPDH antibody (Santa Cruz), followed by HRP-conjugated goat anti-rabbit antibody (Santa Cruz) or goat anti-mouse antibody (Southern Biotech). The signals were developed by chemiluminescence (Pierce).

### Binding ability of SAP to DNA

The binding ability of SAP to DNA was detected by dot blot analysis with mouse SAP protein purified from pSAP treated mice and rabbit anti-mouse SAP (Calbiochem) as previously described [38]. In briefly, DNA (1 µg) was spotted on the nitrocellulose membranes. After the incubation of SAP protein (1 µg/ml), anti-SAP Abs, and peroxidase-labeled IgG Abs (Southern Biotech), the blots were developed with 3, 3′-Diaminobenzidine (DAB) to measure the binding ability of SAP to DNA. Quantitative analysis of blots was done using Mini-Transilluminator (Bio-Rad) equipped with molecular analysis software. The binding ability of SAP to DNA was reflected as mean intensity.

### DNA uptake *in vitro*

ALD-DNA was labeled with Alexa Fluor 488 (Invitrogen) according to the manufacturer's instructions. The labeled ALD-DNA (referred as AF488-ALD-DNA) was purified using Bio-Rad Micro Bio-Spin P-30 column (Bio-Rad, Hercules, CA) according to the manufacturer's protocol. AF488-ALD-DNA was incubated with purified mouse SAP protein (SAP plus ALD-DNA) at 37°C for 2 h. BMDMs were treated with chloroquine (100 µg/ml) before DNA incubation. The intracellular Alexa Fluor 488 labeled ALD-DNA (AF488-ALD-DNA) was determined by flow cytometry (FACSCalibur) as previously described [39]. All flow cytometry data were acquired on a BD FACSCalibur (BD Biosciences) in CellQuest (BD Biosciences) and analyzed by FlowJo software (Tree Star).

### Real-time PCR analysis

Total RNA was isolated from cultured cells with TRIzol reagent (Invitrogen) and was reverse-transcribed (RT) using a cDNA synthesis kit (MBI Ferments) according to the manufacturer's instructions. Subsequently, cDNA was subjected to quantitative real-time PCR using a Lightcycler480 and SYBR Green system (Roche Diagnostics) following the manufacturer's protocol [40].

### Flow cytometry analysis

Murine renal tissues were surgical resected and dispersed in RPMI 1640 contained 5% FBS and 0.1% collagenase (Sigma-Aldrich) at 37°C for 30 min, followed by progressive sieving to obtain single-cell suspensions. To assess the infiltration of leucocyte populations in kidneys of mice, flow cytometry analysis were performed with PE-labeled anti-CD45, PerCP-labeled anti-CD4, FITC-labeled anti-CD19, and FITC-labeled anti-F4/80 (BD Biosciences). All flow cytometry data were acquired on a BD FACSCalibur (BD Biosciences) in CellQuest (BD Biosciences) and analyzed by FlowJo software (Tree Star).

### ELISA Assay

To assess protein levels of TNF-α, IL-1β, IL-6, IL-10, IL-12, and MCP-1 in the homogenized kidney tissue and in serum of mice, ELISA assays were performed with relative ELISA Kits (eBioscience) according to the manufacturer's instructions.

### Pathological analysis

For histology analysis, murine renal tissues were surgical resected and fixed in 4% paraformaldehyde (Sigma-Aldrich), processed, and embedded in paraffin. H&E staining of renal tissue sections were performed according to the manufacturer's instructions and assessed by a pathologist blinded to treatment group. The kidney score of glomerulonephritis was determined by using the ISN/RPS2003 classification. Fluorescent staining of cryosections was used for autoantibody deposition analysis in the glomeruli. Sections were fixed in acetone for 10 min and incubated with FITC-conjugated goat anti-mouse IgG (H+L chain specific) Ab (Sigma-Aldrich) for 30 min. Pictures were acquired with Nikon SCLIPSS TE2000-S microscope (Nikon) equipped with ACT-1 software (Nikon). Original magnification was ×200.

### Statistical analysis

All data are expressed as means ± SD of three independent experiments or from a representative experiment of three independent experiments. The statistical significance of the differences in the experimental data was valued by the Student's t-test. The statistical significance level was set as * *P*<0.05, ** *P*<0.01, *** *P*<0.001.

## References

[1] Rahman A, Isenberg DA (2008). Systemic lupus erythematosus.. N Engl J Med.

[2] Walport MJ (2000). Lupus, DNase and defective disposal of cellular debris.. Nat Genet.

[3] Kotzin BL (1996). Systemic lupus erythematosus.. Cell.

[4] Savill J, Dransfield I, Gregory C, Haslett C (2002). A blast from the past: clearance of apoptotic cells regulates immune responses.. Nat Rev Immunol.

[5] Hoffmann MH, Trembleau S, Muller S, Steiner G (2010). Nucleic acid-associated autoantigens: pathogenic involvement and therapeutic potential.. J Autoimmun.

[6] Qiao B, Wu J, Chu YW, Wang Y, Wang DP (2005). Induction of systemic lupus erythematosus-like syndrome in syngeneic mice by immunization with activated lymphocyte-derived DNA.. Rheumatology (Oxford).

[7] Wen ZK, Xu W, Xu L, Cao QH, Wang Y (2007). DNA hypomethylation is crucial for apoptotic DNA to induce systemic lupus erythematosus-like autoimmune disease in SLE-non-susceptible mice.. Rheumatology (Oxford).

[8] Zhang W, Xu W, Xiong S (2010). Blockade of Notch1 signaling alleviates murine lupus via blunting macrophage activation and M2b polarization.. J Immunol.

[9] Garlanda C, Bottazzi B, Bastone A, Mantovani A (2005). Pentraxins at the crossroads between innate immunity, inflammation, matrix deposition, and female fertility.. Annu Rev Immunol.

[10] Bottazzi B, Doni A, Garlanda C, Mantovani A (2010). An integrated view of humoral innate immunity: pentraxins as a paradigm.. Annu Rev Immunol.

[11] Lu J, Marnell LL, Marjon KD, Mold C, Du Clos TW (2008). Structural recognition and functional activation of FcgammaR by innate pentraxins.. Nature.

[12] Mold C, Gresham HD, Du Clos TW (2001). Serum amyloid P component and C-reactive protein mediate phagocytosis through murine Fc gamma Rs.. J Immunol.

[13] Pepys MB, Butler PJ (1987). Serum amyloid P component is the major calcium-dependent specific DNA binding protein of the serum.. Biochem Biophys Res Commun.

[14] Breathnach SM, Kofler H, Sepp N, Ashworth J, Woodrow D (1989). Serum amyloid P component binds to cell nuclei in vitro and to in vivo deposits of extracellular chromatin in systemic lupus erythematosus.. J Exp Med.

[15] Bickerstaff MC, Botto M, Hutchinson WL, Herbert J, Tennent GA (1999). Serum amyloid P component controls chromatin degradation and prevents antinuclear autoimmunity.. Nat Med.

[16] Bharadwaj D, Mold C, Markham E, Du Clos TW (2001). Serum amyloid P component binds to Fc gamma receptors and opsonizes particles for phagocytosis.. J Immunol.

[17] Triantafyllopoulou A, Franzke CW, Seshan SV, Perino G, Kalliolias GD (2010). Proliferative lesions and metalloproteinase activity in murine lupus nephritis mediated by type I interferons and macrophages.. Proc Natl Acad Sci USA.

[18] Schiffer L, Bethunaickan R, Ramanujam M, Huang W, Schiffer M (2008). Activated renal macrophages are markers of disease onset and disease remission in lupus nephritis.. J Immunol.

[19] Hale MB, Krutzik PO, Samra SS, Crane JM, Nolan GP (2009). Stage dependent aberrant regulation of cytokine-STAT signaling in murine systemic lupus erythematosus.. PLoS One.

[20] Bygrave AE, Rose KL, Cortes-Hernandez J, Warren J, Rigby RJ (2004). Spontaneous autoimmunity in 129 and C57BL/6 mice-implications for autoimmunity described in gene-targeted mice.. PLoS Biol.

[21] Gillmore JD, Hutchinson WL, Herbert J, Bybee A, Mitchell DA (2004). Autoimmunity and glomerulonephritis in mice with targeted deletion of the serum amyloid P component gene: SAP deficiency or strain combination?. Immunology.

[22] Tamaoki T, Tezuka H, Okada Y, Ito S, Shimura H (2005). Avoiding the effect of linked genes is crucial to elucidate the role of Apcs in autoimmunity.. Nat Med.

[23] Napirei M, Karsunky H, Zevnik B, Stephan H, Mannherz HG (2000). Features of systemic lupus erythematosus in Dnase1-deficient mice.. Nat Genet.

[24] Zykova SN, Tveita AA, Rekvig OP (2010). Renal Dnase1 enzyme activity and protein expression is selectively shut down in murine and human membranoproliferative lupus nephritis.. PLoS One.

[25] Murray LA, Rosada R, Moreira AP, Joshi A, Kramer MS (2010). Serum amyloid P therapeutically attenuates murine bleomycin-induced pulmonary fibrosis via its effects on macrophages.. PLoS One.

[26] Fenton K, Fismen S, Hedberg A, Seredkina N, Fenton C (2009). Anti-dsDNA antibodies promote initiation, and acquired loss of renal Dnase1 promotes progression of lupus nephritis in autoimmune (NZBxNZW)F1 mice.. PLoS One.

[27] Emsley J, White HE, O'Hara BP, Oliva G, Srinivasan N (1994). Structure of pentameric human serum amyloid P component.. Nature.

[28] Sorensen IJ, Holm Nielsen E, Schroder L, Voss A, Horvath L (2000). Complexes of serum amyloid P component and DNA in serum from healthy individuals and systemic lupus erythematosus patients.. J Clin Immunol.

[29] Muehrcke RC, Kark RM, Pirani CL, Pollak VE (1957). Lupus nephritis: a clinical and pathologic study based on renal biopsies.. Medicine (Baltimore).

[30] Kuroiwa T, Lee EG (1998). Cellular interactions in the pathogenesis of lupus nephritis: the role of T cells and macrophages in the amplification of the inflammatory process in the kidney.. Lupus.

[31] Andrews BS, Eisenberg RA, Theofilopoulos AN, Izui S, Wilson CB (1978). Spontaneous murine lupus-like syndromes. Clinical and immunopathological manifestations in several strains.. J Exp Med.

[32] Ito T, Schaller M, Hogaboam CM, Standiford TJ, Sandor M (2009). TLR9 regulates the mycobacteria-elicited pulmonary granulomatous immune response in mice through DC-derived Notch ligand delta-like 4.. J Clin Invest.

[33] Lake FR, Noble PW, Henson PM, Riches DW (1994). Functional switching of macrophage responses to tumor necrosis factor-alpha (TNF alpha) by interferons. Implications for the pleiotropic activities of TNF alpha.. J Clin Invest.

[34] Sheerin NS, Abe K, Risley P, Sacks SH (2006). Accumulation of immune complexes in glomerular disease is independent of locally synthesized c3.. J Am Soc Nephrol.

[35] Chintalacharuvu SR, Wang JX, Giaconia JM, Venkataraman C (2005). An essential role for CCL3 in the development of collagen antibody-induced arthritis.. Immunol Lett.

[36] Dupont KM, Sharma K, Stevens HY, Boerckel JD, Garcia AJ (2010). Human stem cell delivery for treatment of large segmental bone defects.. Proc Natl Acad Sci U S A.

[37] Xu J, Yun X, Jiang J, Wei Y, Wu Y (2010). Hepatitis B virus X protein blunts senescence-like growth arrest of human hepatocellular carcinoma by reducing Notch1 cleavage.. Hepatology.

[38] Estabrook MM, Jack DL, Klein NJ, Jarvis GA (2004). Mannose-binding lectin binds to two major outer membrane proteins, opacity protein and porin, of Neisseria meningitidis.. J Immunol.

[39] Chung EY, Liu J, Homma Y, Zhang Y, Brendolan A (2007). Interleukin-10 expression in macrophages during phagocytosis of apoptotic cells is mediated by homeodomain proteins Pbx1 and Prep-1.. Immunity.

[40] Gao B, Duan Z, Xu W, Xiong S (2009). Tripartite motif-containing 22 inhibits the activity of hepatitis B virus core promoter, which is dependent on nuclear-located RING domain.. Hepatology.

